# A materials data framework and dataset for elastomeric foam impact mitigating materials

**DOI:** 10.1038/s41597-023-02092-4

**Published:** 2023-06-05

**Authors:** Alexander K. Landauer, Orion L. Kafka, Newell H. Moser, Ian Foster, Ben Blaiszik, Aaron M. Forster

**Affiliations:** 1 National Institute of Standards and Technology, Material Measurement Laboratory, 100 Bureau Drive, Gaithersburg, MD 20899 USA; 2 National Institute of Standards and Technology, Material Measurement Laboratory, 325 Broadway, Boulder, CO 80305 USA; 3 Data Science and Learning Division, Argonne National Laboratory, 9700 S. Cass Avenue, Lemont, IL 60439 USA; 4 Department of Computer Science, University of Chicago, 5801 S. Ellis Ave., Chicago, IL 60637 USA; 5 Globus, University of Chicago, 5801 S. Ellis Ave., Chicago, IL 60637 USA

**Keywords:** Mechanical engineering, Polymers, Mechanical properties, Biomedical engineering

## Abstract

The availability of materials data for impact-mitigating materials has lagged behind applications-based data. For example, data describing on-field helmeted impacts are available, whereas material behaviors for the constituent impact-mitigating materials used in helmet designs lack open datasets. Here, we describe a new FAIR (findable, accessible, interoperable, reusable) data framework with structural and mechanical response data for one example elastic impact protection foam. The continuum-scale behavior of foams emerges from the interplay of polymer properties, internal gas, and geometric structure. This behavior is rate and temperature sensitive, therefore, describing structure-property characteristics requires data collected across several types of instruments. Data included are from structure imaging via micro-computed tomography, finite deformation mechanical measurements from universal test systems with full-field displacement and strain, and visco-thermo-elastic properties from dynamic mechanical analysis. These data facilitate modeling and design efforts in foam mechanics, e.g., homogenization, direct numerical simulation, or phenomenological fitting. The data framework is implemented using data services and software from the Materials Data Facility of the Center for Hierarchical Materials Design.

## Background & Summary

**Fig. 1:**
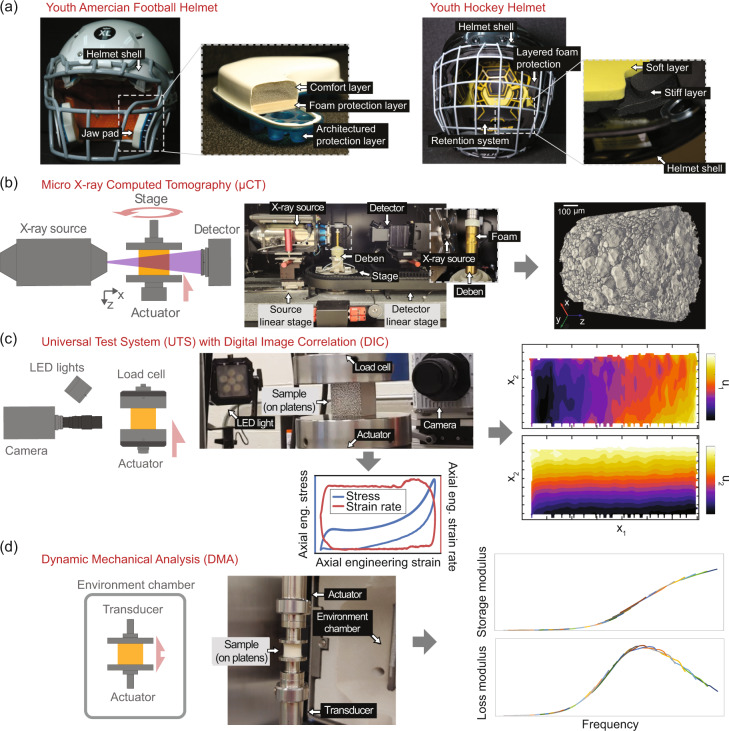
Schematics and photographs of equipment for the dataset and example data. (**a**) Examples of helmet liner applications for impact protection foams. *Left* A youth American football helmet with inset highlighting the multilayered pads that include two foams, a comfort layer and a stiffer vinyl nitrile foam protective sub-layer, above an architectured thermoplastic polyurethane backer. *Right* A youth hockey helmet with inset highlighting softer and stiffer impact protection foam layers. (**b**) Micro X-ray computed tomography laboratory-scale system, where the specimen is placed either on a freestanding platen (not shown) or inside the Deben load tube for *in-situ* compression (see *inset*). In either case these ride on a rotating stage and sequential exposures are taken. This allows the use of a tomographic projection algorithm to reconstruct a volumetric image (shown at right) of the specimen. (**c**) Displacement-controlled load frame with 2D digital image correlation system to measure stress-strain (shown at bottom) with full-field displacement (shown at right) and strain measurements at a range of applied strain rates. (**d**) Dynamic mechanical analysis using a paired-type transducer-actuator for small amplitude oscillatory strain, with the specimen epoxied between platens inside an environmental chamber, from which shifted time-temperature superposition data (shown at right) can be obtained.

Materials databases for physical properties in material science have been a driving factor in enabling big-data approaches to material discovery, characterization, and modeling^1–4^. For protective materials applications, such as helmets, the biomechanics of impact have led the way in data collection and availability, for example, in on-field impact data^5^). Foams or architectured systems (e.g., honeycombs^6^ or controlled buckling structures^7,8^) usually constructed from a polymer-based matrix material serve as protective, energy dissipating layers. The two youth sports helmets in Fig. 1a show examples of multilayered approaches with foam and thermoplastic urethane (Fig. 1a*left*) and a layered foam with soft retention system (Fig. 1a*right*). Polymer properties, gas dynamics, and geometric structure affect protection by limiting energy transfer during impact. Structure-property relationships are critical for defining and modeling performance characteristics of these layers, and, hence, the complete protective system.

The availability of materials information and material property datasets for polymer foams is limited. Occasionally, raw and processed materials data have been provided to support a specific publication, e.g., Landauer *et al*.^9^; however, the vast majority of polymer foam mechanics work has used the anachronistic closed-data, closed-code model, where accompanying datasets and model infrastructure are omitted or inaccessible, rather than open-data and open-source models that promote scientific repeatability^10,11^ and facilitate advancements in the field^12^. Data from commercial manufacturers of these materials are often restricted to standards-based empirical test methods such as indentation force deflection (American Society of Testing Materials, Inc., (ASTM) Standard D5672) or compression set (ASTM D395) and basic measurements such as hardness (ASTM 2240) or tensile strength (ASTM D412). These test methods provide standardized comparative or screening methods for specific, narrow applications or measurements. In general, the physical properties data available in technical descriptions of polymer foams are largely unsuitable for material modeling or higher level structure-property knowledge – activities of increasing demand as design and innovation of protective equipment relies on quantitative modeling and computational design methods^13^. Part of the data and modeling challenge is due to the variety of base polymers and processing techniques used to make foams, and the diverse behaviors that arise from combining non-linear matrix materials with random, heterogeneous, and often anisotropic meso-structures. The resultant material characteristics are often poorly described by single-point or single-rate measurements of a given quantity of interest, and thus additional data are needed beyond those used for more conventional engineering materials.

As a step towards building a communal asset for domain knowledge that fosters data sharing within the foam mechanics community, we propose and demonstrate a prototype FAIR^12^ (findable, accessible, interoperable, reusable) data framework for structure and property data with example data for one elastic closed-cell polymer foam that is used to describe the meso-structure, viscoelastic properties, and compression properties for impact protection foams, and that is easily extensible to additional data sources or materials. The internal geometry of a foam gives rise to anisotropy, inhomogeneities, and unique continuum-scale constitutive behavior, which emerges from the interplay of polymer properties, internally contained gas, and geometric structure^14–17^. The continuum behavior is rate and temperature sensitive, therefore, the data to describe these materials is multimodal. Thus, property data from a variety of different sources is often required for modeling efforts to capture the complete response, for example, micromechanical homogenization^18–20^, direct numerical simulation^21,22^, or phenomenological fitting^9,16,23,24^. The included data are from a variety of imaging and force-sensing instruments under a wide range of experimental conditions, and the framework is designed and constructed to be easily extensible and interoperable with new materials and data sources. The interoperable framework is implemented using data services and software provided by the Materials Data Facility^25^ component of the National Institute of Standards and Technology (NIST) Center for Hierarchical Materials Design.

We demonstrate this framework using a dataset obtained from a closed-cell vinyl-nitrile impact protection foam. These data, with metadata and documentation, include structure data from X-ray micro-computed tomography (*μ*CT), load frame stress-strain data for quasi-static up to impact-like compressive engineering strain rates with full-field displacement data from digital image correlation (DIC), and small amplitude oscillatory strain dynamic mechanical analysis (DMA) data including time-temperature superposition (TTS) shifted frequency curves of storage modulus (*E*′), loss modulus (*E*″), and loss tangent (tan(*δ*) = *E*″/*E*′). Figure 1b-d schematically and pictorially describes the test equipment and shows example visualizations of data. Example code to extract and operate on the data is also provided in a separate GitHub repository (see the Data interfaces section). To improve accessiblity, interoperablity and reusablity, the datasets within the framework consist of experimental metadata, raw machine data (often in proprietary formats), exported data in common, widely used, or open formats, and example post-processed data. We believe these datatypes represent the backbone of materials data required for many analyses and models and welcome and invite new data types, dataset contributions, and suggestions, and feedback from the community to improve the breadth and depth of FAIR data available for foams.

## Methods

### Sample preparation

The initial example case for the data framework is a commercially available vinyl-nitrile closed-cell impact protection foam with a density approximately 110 kg/m^3^ (Impax VN600, Dertex Corp., Saco, DE [see Acknowledgements section]) that we refer to as “VN01”. Three specimen geometries were used: approximately 9 mm diameter by 10 mm height cylinders for *μ*CT, approximately 6 mm diameter by 10 mm height cylinders for DMA, approximately 32 mm by 32 mm by 19 mm rectangular cuboids for quasi-static compression, and approximately 45 mm by 45 mm by 25 mm rectangular cuboids for intermediate rate load frame experiments. The cylindrical samples were excised from an as-received approximately 10 mm thick skived sheet using a graphite-lubricated brass cutting die in a low-speed manual drill-press. Cuboidal specimens were cut with a vertical bandsaw from as-received, approximately 50.4 mm thick skived sheets. Specimen surfaces used for DIC were first airbrushed with a thin layer of white paint, then stencil patterned with speckles consisting of dot diameters of either 0.33 mm (for the high resolution cameras) or 0.66 mm (for high-speed cameras). Specimen details are summarized in Table 1.

**Table 1:** Specimen provenance, analysis applied, and data collected for each experiment described in the dataset.

Test type	Material type	Sheet	Specimen Number	Analysis	Data collected
*μ*CT	VN600	D06	S001, S002, S003 and S004	Binarization	Raw images, image volumes, binarized volumes, relative density
Quasi-static rate UTS	VN600	001	003	2D-DIC	Stress and displacement, DIC images, DIC displacement and strain
Intermediate rate UTS	VN600	001	Primary: 03 Validation: 06, 07, 08	2D-DIC	Stress and displacement, DIC images, DIC displacement and strain
DMA	VN600	D06	Primary: 03 and 04 Validation: 01, 02, 05	Time-temperature shifting	Storage and loss moduli, shift factors, shifted curves

### Micro-computed tomography

For μCT two test modes were used: static high-resolution imaging and *in-situ* compression series at reduced resolution. In both cases, specimens were fixed to the instrument platen(s) with double-sided adhesive tape. Since good lubrication is not practical in the *in-situ* compression fixture and transverse strains are relatively small constraining the specimen improved repeatability. After mounting, the specimens were left, unloaded, in the X-ray chamber (with the beam off) for approximately 24 h to allow thermal equalization with the roughly 28 °C chamber, and to allow for mechanical relaxation after handling. The waiting period enabled sharper images than could be obtained without a waiting period, since small motions of the microstructure that occur during equilibration result in blur.

**Fig. 2:**
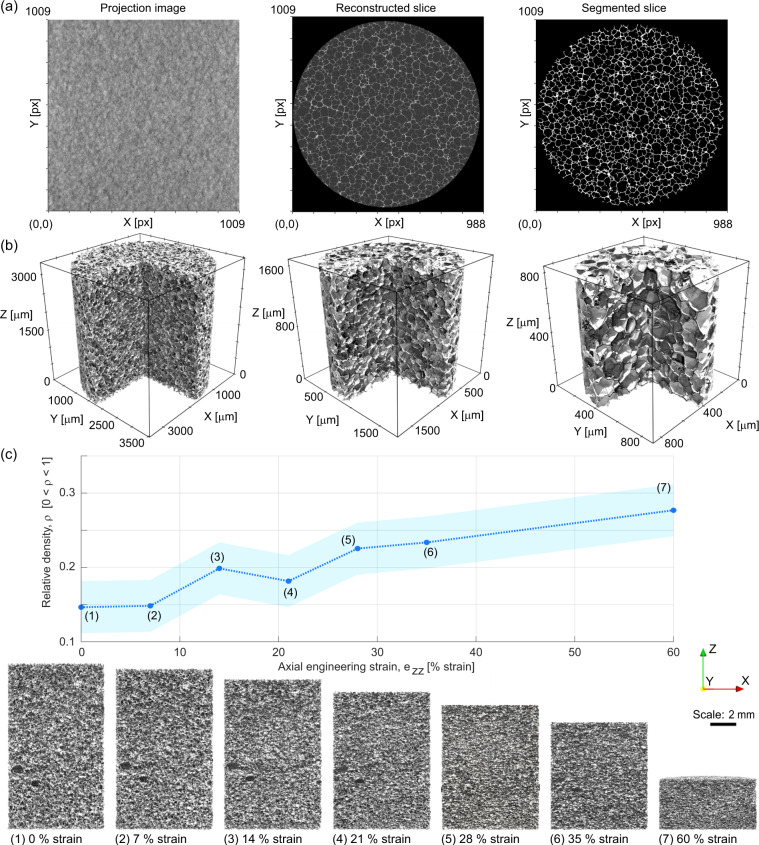
Examples of data included in the μCT dataset. (**a**) Example raw projection image, a grayscale reconstruction of a horizontal slice, and a different binarized horizontal slice. (**b**) 3D renderings of the three “baseline” high-quality μCT images, with increasing resolution (higher magnification) left to right. (**c**) Example usage of *in-situ*μCT. The plot on the top shows relative density computed from the lower resolution (“4X02”) segmented 3D images plotted against applied strain, showing a generally increasing trend in density as compressive engineering strain increases from 0 % strain to 60 % strain (the variation from this trend is likely due to generally low contrast images and variations in imaging settings necessitated by the applied compression). The shaded region shows the standard uncertainty derived from a sensitivity analysis on key segmentation parameters. The images below the plot show vertical slices of the reconstructed volume image of the foam at each strain step. These images show the center plane of each specimen. They can be used to, for example, identify morphology and deformation habits of specific features within the 3D structure or investigate inhomogeneities (e.g., compaction banding) during the deformation.

Polymer foams provide relatively low contrast for the hard X-rays typically used in laboratory-scale X-ray microcomputed tomography. To overcome this challenge, relatively low X-ray voltage (50 kV) was paired with relatively long exposure time (20 s to 30 s per projection) to obtain radiographs with sufficient contrast to enable 3D reconstruction. Each μCT “scan” consists of at least 1601 radiograph projections (see Fig. 2a) collected over a 360° rotation. This resulted in scans lasting about 24 h each. These were reconstructed into 3D volumetric images by using the proprietary Zeiss software, Scout-and-Scan Reconstructor version 14 (Fig. 2a). The reconstructions were exported to 16-bit grayscale TIFF (i.e., .tiff file format) image stacks, which are widely operable with open source software.

Custom Python software within the Anaconda environment (see: https://anaconda.com) was used to process and binarize (segment) the 3D grayscale images^26^. A series of 2D and 3D filters were employed: first, a 2D slice-wise non-local means denoising filter was applied, followed by a 3D unsharp mask. Then, hysteresis thresholding (as implemented by SciKit-Image v. 0.19.0^27^), was applied, resulting in a black-and-white segmented image stack. If required, a morphological opening or closing operation was applied, depending on the specifics of the image stacks: see Table 2, as well as Figs. 2a and 6a. The resulting stack of 2D slices represents the 3D image as a segmented (binary) volumetric image of the foam (for example, see Fig. 2a).

**Table 2:** Specifications and settings for typical μCT datasets.

Image category	4× baseline	8× baseline	16× baseline	4× with *in situ* compression	20× with *in situ* compression
Source voltage [kV]	50	50	50	40	40
Source amperage [A]	80	80	80	75	80
Pixel size [m]	3.60 ± 0.08	1.80 ± 0.04	0.900 ± 0.02	4.00 ± 0.09	0.66 ± 0.02
Number of projections	3001	3001	2401	3201	1801
Number of z-slices	995	991	976	1856	970
Size of image slices [px]	988 by 1009	988 by 1009	984 by 1009	987 by 1009	987 by 1009
Non-local means cut-off	0.01	0.02	0.045	0.015	0.08
Non-local means patch size	5	7	9	9	4
Non-local means search distance [px]	5	7	7	9	5
Unsharp mask kernel radius [px]	1	1	2	2	N/A
Unsharp mask strength	1	1	0.5	1	N/A
Hysteresis threshold, lower [graylevel]	48	52	98	43	191
Hysteresis threshold, upper [graylevel]	58	62	108	51	231
Binary closing disk radius [px]	1	1	2	1	N/A

Thorough descriptions and complete listings of specifications and settings, including uncertainties on measurands, can be found in the documentation and notes in the respective Python methods and parameters files.

After segmentation, 3D voxel models of the cellular structures were created. For visualization, the images were downscaled by a factor of two in all dimensions before the 3D voxel models were generated, which saves computational resources (memory) without substantial loss of information. The sharp edges of voxels can be difficult to render and visualize for complex structures due to the presence of abrupt shadows. Thus, the voxel models were also converted to surface meshes using the Marching Cubes algorithm implemented in SciKit-Image v. 0.19.0^27^. The resultant triangle-based surface-meshes were then exported into a data format suitable for ParaView^28^ (i.e., .stl or .vtk) via The Visualization Toolkit^29^ (VTK) open-source library. Digital-rendering examples of these surface meshes using ParaView are given in Figs. 2b and 6a.

For the compression series, an *in-situ* load frame (see Fig. 1b) was used to impose static compressive strains on the foam during imaging. The general X-ray imaging method for the compressed foam is largely the same as described above, except that between each subsequent volume image the applied displacement was increased at a rate of 0.2 mm/min until the next prescribed strain level was reached. At each step displacement was held constant for the scan. Before each scan, a wait period was applied – primary relaxation was allowed to proceed before imaging (generally around 1 h, although each hold was determined based on stabilisation of the force readback rather than a fixed amount of time). Two *in-situ* compression experiments were conducted at different resolutions, one at high resolution with 0.662 μm/voxel-edge and one at lower resolution of 4.00 μm/voxel-edge. The high resolution specimen was imaged at 0 % strain, 20 % strain, 30 % strain, 40 % strain, 50 % strain, and 70 % strain. The low resolution specimen was imaged at 0 % strain, 7 % strain, 14 % strain, 21 % strain, 28 % strain, 35 % strain, and 60 % strain. The evolution of the binarized view and resultant relative density as a function of increasing *in-situ* uniaxial compressive strain as derived from *in-situ* mechanical testing with μCT at lower resolution are shown in Fig. 2c. These data enable conduction of further analyses, such as anisotropy evolution described in^17^ or digital volume correlation.

### Quasi-static rate uniaxial compression

**Fig. 3:**
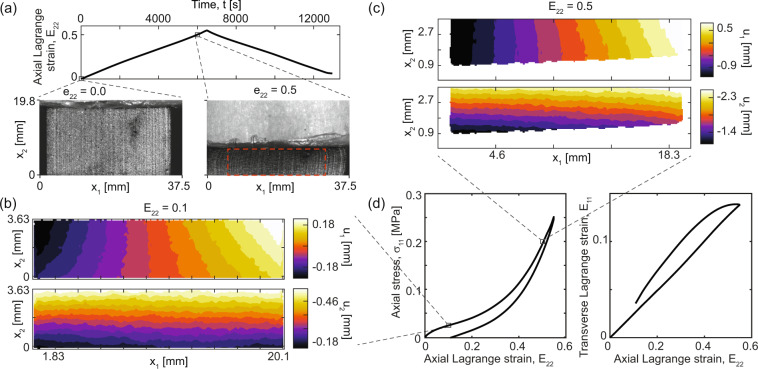
Example data from a single typical quasi-static rate experiment with 2D-DIC. (**a**) Time-history of the mean axial Lagrangian strain (*E*_22_), with example images selected at zero compression and 50 % compressive strain. The red dashed box indicates the region of interest used for DIC. (**b**) Example 2D-DIC displacement fields (*u*_1_, top, and *u*_2_, bottom) on the specimen surface at approximately 10 % axial strain. Note that *u*_1_ is nominally symmetric about the vertical centerline and the *u*_2_ component is minimum at the bottom edge and maximum at the top edge, since the uniaxial compression is applied between a fixed top platen and moving bottom platen. (**c**) Analogously to (**b**), the 2D-DIC displacement fields (*u*_1_, top, and *u*_2_, bottom) on the specimen surface at approximately 50 % axial strain, showing a small amount of frictional confinement on the upper platen in *u*_1_. (**d**) The axial engineering stress and mean transverse Lagrangian strain plotted against the mean axial Lagrangian strain for one example. Standard uncertainty on stress is circa ±0.18 kPa.

Uniaxial compression experiments were conducted using an electromechanical universal test system (UTS) with a nominally 500 N load cell at a quasi-static strain rate. We define a “quasi-static” rate to be a strain rate slow enough that rate-dependent viscous effects are negligible, i.e., any hysteresis present is not rate-dependent. Full-field displacement and strain were captured using 2D-DIC measurements. DIC images were collected with a high-resolution (8.9 megapixel, grayscale 8-bit CMOS) scientific camera (FLIR Blackfly S, Richmond, BC, Canada) triggered from the start-of-test signal from the UTS. The sample was illuminated with two high-intensity light emitting diode light sources (3200 lumens each, 10° beam angle) with vertical polarizers. A low magnification telecentric lens (TitanTL 0.377×, 22.5 mm field of view) was used to minimize the parasitic strain effects due to out-of-plane motion with a horizontal polarization filter to eliminate specular reflections deleterious to DIC accuracy^30,31^. A list of specifications and typical DIC parameters are given in Table 3. Force was measured with a calibrated 500 N tension/compression force transducer with force resolution 8.9 mN and within a ±0.25 % force error throughout the full scale range. A series of three experiments on a single representative specimen were conducted after confirming that specimen-to-specimen variability in force-displacement response was negligible. Experiments were conducted under ambient conditions with temperatures in the range of (22.6, 24.3) °C and at a quasi-static engineering strain rate of 10^−4^ s^−1^ using a triangular displacement-time profile. Collecting the complete load-unload cycle is important to obtain the complete stress-strain response of the specimen including viscous effects. This is particularly important for elevated rate testing, but in nominally quasi-static experiments can reveal, for example, the quasi-rate-independent hysteresis in Fig. 3d. The complete load-unload history can be useful in diagnosing experimental shortcomings or planning model fitting routines (e.g., in^9^). The specimen loading surfaces in contact with the compression platens were lubricated first with graphite powder and immediately prior to each experiment with a thin layer of high-pressure multi-purpose lithium grease. The camera frame rate was 0.1 Hz (approximately 80 ms exposure time) and force sampling was 1 Hz. Force was downsampled to match the strain imaging rate when generating stress-strain data.

**Table 3:** Digital image correlation specification details for the quasi-static and intermediate rate cases.

Parameter	Quasi-static compression	0.01 s^−1^ compression	0.10 s^−1^ compression	1.0 s^−1^ compression	10 s^−1^ compression
Technique	Single camera q-factor Based Digital Image Correlation^32^				
Lens	TitanTL 0.377× 22.5 mm telecentric	Sigma 105 mm Macro			
Pre-filtering	Gaussian, [3px, 0.5]				
Min. Subset Size [px]	32	16			
Min. Step Size [px]	8	2			
Correlation criterion	Iterative FFT-based ZNCC				
Strain measurement	Global plane fitting	9-tap optimal spatial differentiation filter			13-tap optimal differentiation filter
Interpolation	Bicubic splines				
Camera noise [% full scale range]	0.54	1.95	0.36	0.53	0.21
Image size (approx.) [px by px]	1900 × 350	400 × 100	320 × 100	320 × 100	400 × 100
Measurement points	39600	2825	2825	2825	7889
Number of images	650 (0.05 Hz)	188 (6 Hz)	200 (60 Hz)	344 (500 Hz)	201 (1500 Hz)
Pixel-to- μm conversion	1 px = 9.15 μm	1 px = 112 μm	1 px = 112 μm	1 px = 112 μm	1 px = 112 μm
Noise floor [px]	0.016	0.014	0.016	0.028	0.015
Virtual strain gauge [mm]	ca. 18	5.38	5.38	5.38	7.77

The data for each column are drawn from a single example, usually the first run in the dataset, and are typical approximates. For more detailed interpretations of each parameter see Landauer *et al*. 2018^32^ and more details for each experimental case are available in the full dataset.

For full field strain and displacement from the images, a free and open source DIC algorithm^32^ was used. Custom pre- and post-processing for the UTS data are included with the DIC scripts to combine displacement and strain data with crosshead (time, force) measurements. The full-field displacement data consists of 2D displacement vectors in the *x*_1_ and *x*_2_ directions organized on a regular grid with mesh spacing of 8 px. For this configuration, a whole-image plane-fitting routine with multiplicative decomposition of the strain tensor^9^ is used to compute total axial (*E*_22_) and transverse (*E*_11_) Lagrangian strains. Other methods to compute full strain maps may be employed by adapting the scripts described below for the intermediate rate case.

### Intermediate rate uniaxial compression

**Fig. 4:**
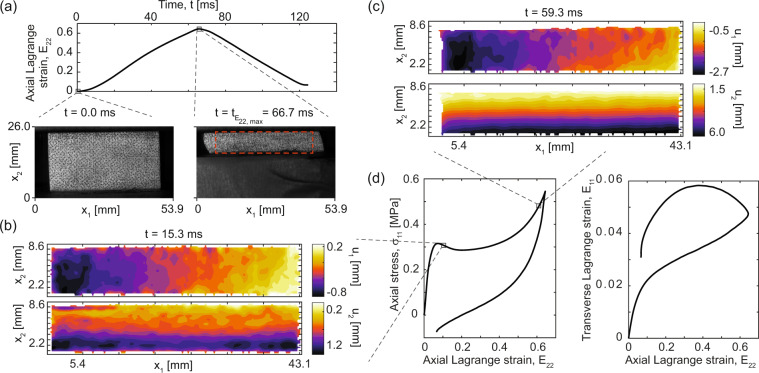
Example data from a single typical intermediate rate compression experiment at the fastest rate (nominally 10 s^−1^ or equivalently 250 mm/s). (**a**) Time-history of mean axial Lagrangian strain (*E*_22_), with example images selected at zero compression and maximum compression. The red dashed box indicates the region of interest used for DIC. (**b**) Example 2D-DIC displacement fields (*u*_1_, top, and *u*_2_, bottom) on the specimen surface at approximately 15 % mean axial strain. Note that *u*_1_ is nominally symmetric about the vertical centerline and the *u*_2_ component is maximum at the bottom edge and minimum at the top edge, since the uniaxial compression is applied between a fixed top platen and moving bottom platen (in contrast to the quasi-static case). (**c**) Analogously to (**b**), the 2D-DIC displacement fields (*u*_1_, top, and *u*_2_, bottom) on the specimen surface at approximately 60 % mean axial strain, showing a less even and noisier gradient in *u*_2_, which is may be related to the non-monotonicity of the stress-strain curve at this rate and strain level and increased uncertainty associated with the high-speed imaging configuration. (**d**) The axial engineering stress and mean transverse Lagrangian strain plotted against the mean axial Lagrangian strain for one example. Standard uncertainty on stress is circa ±1.1 kPa.

Intermediate strain rate uniaxial stress experiments with 2D-DIC were conducted with a servo-hydraulic UTS. We define intermediate rates to be the strain rates regime where rate-dependent viscous effects are substantial, while inertial effects remain minimal. These elevated strain rate experiments were conducted in a system capable of displacement control at rates in excess of 1 m/s (the fastest 10 s^−1^ rate in this dataset is equivalent to approximately 0.25 m/s crosshead speed) equipped with a calibrated 25 kN-rated tension/compression force transducer with force resolution 2.2 N and a ±0.6 % error throughout the full scale range. A high-speed camera (SA5, Photron USA Inc., San Diego, CA, USA) with full-frame resolution of 1024 px by 1024 px at up to 7000 frames per second (fps) and 20 μm monochromatic pixels was used to record images for 2D-DIC, which was triggered from the UTS. Eight-bit grayscale images were captured with a 105 mm, 1:2.8 macro lens (DG Macro HSM, Sigma Corp, New York, NY; working distance approximately 0.5 m, f/5.6) focused on the specimen surface and illuminated with two high-intensity light emitting diode light sources (3200 lumens each, 10° beam angle). Due to the relatively low resolution and large pixel size of the high-speed cameras the speckle sizes are near the aliasing limit, which introduces additional noise compared to the quasistatic case (see Fig. 4b), and is particularly apparent in transverse strain maps. Experiments were conducted under ambient conditions with temperature in the range (17.3, 24.8) °C. Decadal engineering strain rates were selected from close to quasi-static to impact-like: 10^−2^ s^−1^, 10^−1^ s^−1^, 10^0^ s^−1^, 10^1^ s^−1^ as specified in Table 3 using a triangular displacement-time profile.

As in the quasi-static case, the 2D-DIC data provides full-field displacement vectors on the specimen surface for each time step. In this case, the DIC algorithm^32^ was used with a smaller mesh step of 2 px in conjunction with pre- and post-processing to compute strains via a spatial differentiation kernel^15^. The total strains are computed as in the quasi-static case. Since triggering is subject to hardware delays in the UTS and framing time in the cameras, manual time alignment was used as needed. Due to the timing delay between triggering the UTS and cameras and the start of motion, the the fastest-rate experiments have roughly 320 frames collected prior to motion start. To avoid accumulating error throughout these frames the image and corresponding force data are truncated to times during which motion occurs, and force data is resampled with a spline interpolation to the exact time points when images were captured, thus aligning stress and strain data to a self-consistent time datum. The dataset includes raw images with timestamps referenced to the trigger signal in addition to the UTS output signals and timing, and thus other DIC algorithms or time-alignment can be employed. The DIC parameters used are given in Table 3.

### Dynamic mechanical analysis (DMA)

Dynamic mechanical analysis was conducted on a separate motor-transducer instrument (RSA G2, TA Instruments, New Castle, DE) with cylindrical specimens, as described in the sample preparation section, bonded to compression platens with a rigid two-part epoxy resin. Two types of experiments are included in the dataset: preliminary, frequency sweep, amplitude sweep and temperature equilibration experiments, and, temperature-frequency sweep primary experiments. The preliminary measurements are required to establish that the time-temperature superposition measurements are within the linear viscoelastic region of the foam. These also are used to refine method parameters to optimize instrument sensitivity with respect to material parameters. The strain amplitude and strain rate settings must be below the maximum allowable force limit (approximately 22 N), above the strain and force noise floors (approximately 5.6 μstrain and 0.05 N), below the instrument harmonic frequency (approximately 50 Hz), and within the controllable temperature range (approximately −100 °C and 400 °C). Within the machine constraints, the linear viscoelastic range needs to be established. To do this, the effect of changing material non-linearities with both rate and temperature, the thermal expansion and time to establish thermal equilibrium, and the applicable temperature range and glass transition temperature (*T*_*g*_) regimes – all material characteristics – are characterized using the preliminary experiments to parameterize the time-temperature superposition (TTS) method. These experiments and their resultant key parameters are outlined in Table 4. The methods and analyses were set up and conducted in the proprietary instrument software (Trios Version 5.1.1.46572, TA Instruments, New Castle, DE), and we have included methods and data in the associated proprietary data format. Data are also provided as spreadsheets where applicable.

**Table 4:** Listing of the dynamic mechanical analysis experiment types.

Test type	Test subtype	Target parameters	Temperature range [°C]	Frequency range [rad/s]
Temperature equilibration with oscillation	Fixed amplitude, frequency and temperature	Equilibration times (thermal and viscous)	75, −10	6.28
Strain sweep	Fixed temperature and frequency	Amplitude ranges	25, −10, −60	6.28
Frequency and temperature sweep	TTS data collection	Complete sweep	60 to −35	0.3 to 30

These include a typical set of calibration experiments to establish protocol settings and range for the frequency and temperature sweep series used to build a final time-temperature superposition (TTS) curve. Temperature and frequency specifications are given as nominal values.

**Fig. 5:**
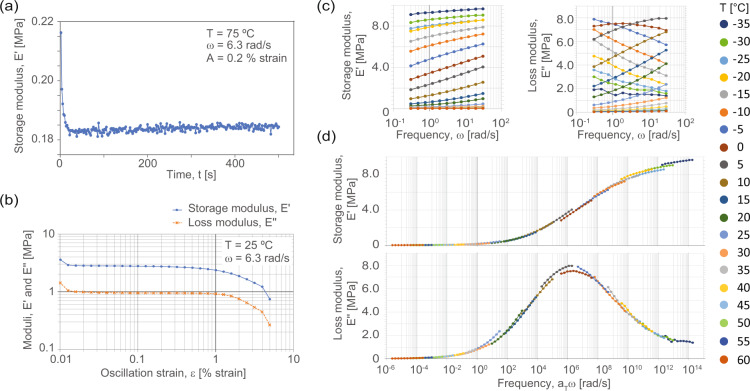
Example data from calibration and time-temperature superposition (TTS) dynamic mechanical analysis (DMA) experiments. (**a**) An example temperature equilibration time experiment showing a decay in small-strain storage modulus that equilibrates in about 50 s. (**b**) A typical oscillation strain amplitude sweep, showing non-linear effects below and above a target zone (0.02 to 0.2) % strain in log-log space. (**c**) Temperature-frequency sweep segments before shifting, as shown at right. (**d**) Complete, shifted frequency sweep data at a reference temperature of 25 °C for this example.

The small amplitude oscillatory strain (SAOS) TTS sweeps were thus taken over a temperature range of nominally −35 °C to 60 °C, sampled at steps of approximately 5 °C or 3 °C depending on experiment and temperature, frequency range of nominally 0.3 rad/s to 30 rad/s with logarithmic sampling with 5 points per decade, and an amplitude range of about 0.05 % strain to 0.39 % strain, which stayed within the linear viscoelastic regime of the stress-strain response. A small precompression force was applied such that the compressive normal force was always greater than 150 % of the maximum dynamic oscillation force, and the pre-strain due to this force was less than approximately 7 % compressive engineering strain. Example data from a subset of calibrations and a resultant full TTS curve at a reference temperature (*T*_*ref*_) of *T*_*ref*_ = 25 °C is shown in Fig. 5.

## Data Records

As described in the Methods section, data were collected from four primary experimental sources: *μ*CT, quasi-static uniaxial compression with 2D-DIC, intermediate rate uniaxial compression with 2D-DIC, and DMA. Both primary raw data from the instruments and post-processed data are collected in the data repository, with a top-level directory for each type of experiment. An archival data repository record for this work is stored on the NIST Public Data Repository (PDR) system^33^, and a live version^34^ with additional functionality for scriptable data access (see the *Data interfaces* section) is implemented and maintained via data services and software provided by the Materials Data Facility (MDF)^25^ component of the NIST Center for Hierarchical Materials Design (https://materialsdatafacility.org). As a hub for materials data co-hosting the MDF improves the findablity of the data. Additionally, the scripting interface encourages easy access, interoperablity with Python data processing routines, and reusablity for the data framework and datasets – example open source scripts showcasing this are provided as Jupyter notebooks.

Details of specimens used for each of the experimental modalities are given in Table 1. Each experiment type directory contains a text file documenting the data framework, directory structure, naming conventions, file types and other details, and a table of contents text file describing each experiment instance alongside sub-directories for each material type and the supporting analysis code. At the time of publication one material type is included, namely the vinyl-nitrile foam described in the sample preparation section and coded VN01.

In the data repository, the “microCT_data” directory contains raw and processed μCT images of the exemplar VN01 foam (sub-directory “VN01”), as well as a set of Python scripts (sub-directory “supporting_analyses”) used to process the 3D image stacks after extracting them from the propriety, machine specific format in which they originate (also included, in “RAW” subfolders, see below). Both directories themselves have sub-directories that can be broadly divided into two categories: 1) baseline, high quality zero displacement (indicated by “BASE” in the folder name) and 2) *in-situ* compression (“COMP”). Basic parameters used for processing each dataset are given in Table 2.

The “*BASE” folders encompass low, medium and high effective magnification volumetric images of the VN01 foam. These include 4× optical magnification, 4× optical and 2× geometric (8× total) magnification, 4× optical and 4× geometric (16× total) magnification. For each magnification, the datasets contain three types of data: RAW, IMG, and BIN. The RAW-type folders are the most fundamental images, proprietary-format projection images (.txrm files) and μCT machine setup files (.rcp and .png). The IMG-type folders contain grayscale images (.tif) with the parameters used for reconstructing volumes from the raw projections (.txt). The BIN-type folders contain binary images stacks (representing 3D images as a series of .tif files) created using the thresholding process described above, a file (.txt) describing the settings used to convert grayscale to binary images, a file containing the computed relative density (portion of white voxels in the region of foam in the binary image, .csv), and compressed (.zip) surface mesh (.stl and .vtk) as well as a 3D voxel model (.vtk) of the binarized volume. Text files (.txt) containing additional notes are used to describe the details of each dataset.

The “*COMP” folders (one for higher resolution images using 20× optical magnification, and one with lower resolution images using 4× optical magnification) contain four types of data: RAW, IMG, BIN, and LOAD. The RAW-type folders are the most fundamental images, proprietary-format projection images (.txrm files in compressed .7z containers) and μCT machine setup files (.rcp). The IMG-type folder contains grayscale images (.tiff or .tif) and the details of data collection and image reconstruction (i.e., a stack of images representing a 3D volume) in machine-readable format (.csv). The BIN-type folders contain binary image-stacks (representing 3D images as a series of.tif files) created using the thresholding process described above, a file (.txt) describing the settings used to convert grayscale to binary images, a file containing the computed relative density (portion of white voxels in the region of foam in the binary image, .csv), and compressed (.zip) representations of surface meshes (.stl and .vtk) and 3D voxel models (.vtk) of the binarized volume. Finally, the LOAD-type folder contains force-displacement and loading rate data collected during *in-situ* compression testing of the foam (.csv). Text files (.txt) containing additional notes are used to describe the details of each dataset.

The folder “quasistatic_rate_data/VN01” contains sub-directories for each quasistatic rate experiment, coded by repeat number (see the included data_framework_quasistatic.txt and VN01_notes_quasistatic.txt files). Each experiment sub-directory contains crosshead data (time, commanded axial displacement, readback axial displacement, and axial force) in .csv format, qDIC results data in a .mat, and a sub-directory containing the complete image sequence for DIC in sequentially numbered .tif images. Processing scripts and documentation to recompute DIC results from the images are included in the “quasistatic_rate_data/supporting_analyses” subfolder. Descriptive text files are include describing the details of each dataset.

Similarly, the “intermediate_rate_data/VN01” directory contains sub-directories for each intermediate rate experiment, coded by nominal strain rate and repeat number. Each experiment sub-directory contains crosshead data (time, commanded axial displacement, readback axial displacement, and axial force) in .csv and .xml formats, qDIC results data in a .mat datastore file, a length scale reference snapshot (.tif), and a sub-directory containing the complete image sequence for DIC in sequentially numbered .tif images. Processing scripts and documentation to recompute DIC results from the images are included in the “intermediate_rate_data/supporting_analyses” subfolder. Descriptive text files are include describing the details of each dataset.

The “DMA_data/VN01” directory contains sub-directories for each of the DMA experiments, coded by a specimen number. Each experiment contains a .tri (Trios native format) data bundle and .xls spreadsheet for each temperature, which include instrument metadata and frequency sweep data for that temperature. Additionally, a sub-directory “*_TTS” contains a combined .xls that includes sheets for metadata and each of the temperatures as individual sheets – this is the format used as input to a Python data preparation script (see Usage Notes) – and a final .xls with the complete data and full shifted TTS, Van Gurp-Palmen, and Williams-Landel-Ferry (WLF) fit curves. Lastly, a “Procedures” sub-directory contains the input procedures for the instrument in the native .tprc format with selected .txt procedure logs). Descriptive text files are included that describe the details of each dataset.

## Technical Validation

Each type of experiment requires checks to establish the validity and accuracy of the resultant data. While complete experimental data for each validation is beyond the scope of the primary data included in example dataset, we describe these considerations and experimental results here.

For the μCT experiments, a series of tuning X-ray images were taken to adjust accelerating voltage, beam power, exposure times, frame averaging, and other imaging settings for each magnification and strain level. After the μCT machine performed automatic calibrations, such as source warm-up and drift correction, the various imaging parameters were manually adjusted to maximize contrast while minimizing noise and imaging artifacts. More specifically, the source power was varied such that the X-ray transmittance was targeted to be approximately 60 % or less, which was necessary to achieve adequate contrast in the X-ray projection images (for *in-situ* images, this was not possible to achieve, but transmittance was minimized to the extent possible). No additional beam filters were necessary to achieve this. Image exposure times were increased to ensure that the scintillation detector measured at least 5000 counts per exposure at the center of the projection images. A vendor-provided reference artifact with geometry known to high precision was used to ensure that the calibration to convert pixels to micrometers deviated by an absolute value of less than 2 %. Moreover, for all of the μCT experiments, at least 1601 projection images were taken while the sample revolved 360°, which was chosen based on the Nyquist sampling criterion. Both the height and width of the projection images were approximately 1000 pixels. The native data type for the X-ray detector is 16-bit, and so, a 16-bit data type was maintained for all of the projection and reconstructed images. Since the cellular ligaments of foams are thin and are primarily composed of low-Z atoms, we did not observe beam hardening in the reconstructed images, and thus, no additional corrections were necessary. Also, during reconstruction, center shift values were chosen to minimize blurring throughout the images.

**Fig. 6:**
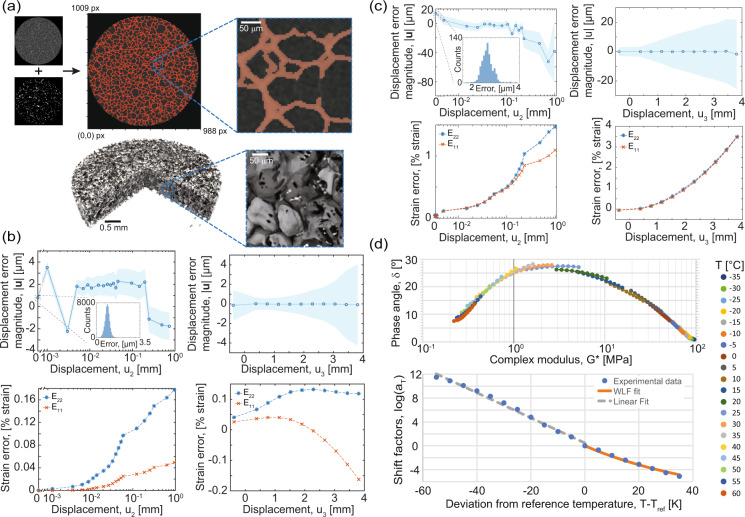
Data validations for the assorted techniques. (**a**) (Top) Example grayscale reconstruction slice with binarization overlay. *Inset* Magnified region to show detail. Some faint features are missed, but overall the binarization accurately traces the material phases seen in the grayscale image, and (bottom) a similar view showing the 3D model based on the binarized image with magnified *inset*. (**b**) Rigid body motion displacement noise for quasi-static rate experiment displacements (top row) and strains (bottom row) in the in-plane (*u*_2_) and out-of-plane (*u*_3_) directions. The first two in-plane motions (0.001 mm and 0.002 mm) are at the displacement noise floor of the actuator. The actuator error dominates the total measurement error. (**c**) Rigid body motion displacement noise for intermediate rate experiment displacements (top row) and strains (bottom row) in the in-plane and out-of-plane directions. For (**b**,**c**) shaded regions represent one standard deviation of displacement above and below the mean. (**d**) (Top) The Van Gurp-Palmen plot of phase angle and (bottom) the Williams-Landal-Ferry and Arrhenius fits of the horizontal shift factors.

The primary concern for binarization of the reconstructed μCT images was avoiding over- or under-segmentation. To accurately segment the images via the thresholding-based method described above, we used a series of sensitivity analyses on the filtering and thresholding parameters, followed by visual inspection of the results. In the sensitivity analyses, a figure of merit, typically relative density, was computed as a function of input segmentation parameter. From these analyses, we found that – during hysteresis thresholding – the grayscale “low” threshold parameter was the strongest parameter in determining relative density, and typically results in an s-curve type of response in the measured relatively density. An acceptable “low” threshold parameter usually occurred at or near the first inflection point, which was visually confirmed by overlaying the binary resultant images over the original grayscale, reconstructed images, as demonstrated in Fig. 6a. A more complete summary of the results are included as a .pdf in the “supporting_analyses” sub-directory.

For the *in-situ* μCT compression experiments, deformation measurements were based on cross-head displacement, measured by an LVDT supplied and calibrated by the load frame vendor. The readback value is given to the closest 1 μm, and the rated displacement resolution cited by the vendor is 3 μm, although no statement of uncertainty on this measurement is provided. Similarly, the load cell was provided as calibrated. The specifics of the calibration were not provided by the vendor. A calibration-mass-based experiment suggested less than 1 % deviation in the load reading throughout the range of loads applied.

Preliminary experiments in the electromechanical universal test system used a nominal strain rate of 10^−2^ s^−1^ in a triangular pulse to 65 % engineering strain. These experiments indicated minimal Mullins-type effects after the first cycle, or other irreversible effects. If specimens are allowed to fully relax (about 0.75 h or longer) between repeats there is acceptable repeatably between specimens, i.e., qualitatively less than typical experimental error sources. Additional experiments conducted in the sheet thickness direction and two mutually orthogonal directions showed evidence of minimal anisotropy (< 10 % difference) in the stress response.

Controlled rigid body motion tests of out-of-plane and in-plane motions using the test specimen demonstrate the respective errors, see Fig. 6. For the 2D-DIC, the out-of-plane motion toward the camera was relatively small (less than 3 mm) compared to the circa 500 mm working distance, such that strain errors are expected to be less than 1 % of strain at maximum. For the quasi-static experiments, parasitic strains due to out-of-plane motion were effectively eliminated by the use of a telecentric lens (Fig. 6b), whereas biaxial parasitic strain is evident in the intermediate rate configuration, see Fig. 6c. However, parasitic strains are small relative to the total strains in these experiments. Rigid body motion errors are at the displacement resolution of the actuator (certified calibration of ±2.5 μm accuracy or better) for the quasi-static case (Fig. 6b), but resolution of the high-speed camera for the intermediate rate experiments is comparably worse, although still low relative to overall displacements (approximately ±20 μm), leading to an increased uncertainty in displacement and strain (Fig. 6c). In general, displacement maps from DIC measurements (see Figs. 3, 4) confirm relatively little off-axis loading (shear or misalignment), barreling due to frictional effects, or strain localization, any of which would indicate a deviation from the assumptions of uniaxial stress. However, a small amount of frictional confinement is apparent particularly during the initial loading stages when the imposed compressive forces are small compared to static friction. We followed the guidance from the International Digital Image Correlation Society’s Good Practices Guide^31^, a community-based standard for DIC practitioners, throughout the DIC experiments.

As described in the Methods section, before collecting and processing time-temperature superposition data and generating shifted final curves, a series of experiments were conducted. These include quantifying time-dependency factors of the material (thermal and strain equilibria) and determining the linear viscoelastic regime. In general, standard uncertainty on the instrument frequency and moduli measurements is estimated to be ±5 % and ±8 %, respectively. The manufacturer specified a standard uncertainty in the temperature as ±0.1 °C. Wait times for strain and temperature equilibration were longer than about 200 s, per the time-dependence in stress measure and shown in Fig. 5a. Data for TTS shifting was collected within the log-linear region of the storage modulus versus amplitude plot, for example as shown in Fig. 5b. The Van Gurp-Palmen plot, for example in Fig. 6d, of phase angle versus complex modulus was generally smooth and consistent with no vertical shifts of the data. Foams are not thermorheologically simple materials, but the experimental method developed here results in sufficient alignment of the frequency-temperature data within the ranges established in preliminary experiments with only horizontal shifting required. Similarly, the fit of the Williams-Landel-Ferry (WLF) equation to the manually optimized shift factors, for example as shown in Fig. 6d, is excellent above T−T_ref_ = 0 K, and a linear fit is good below T−T_ref_ = 0 K. This is the expected range of applicability for polymers. The single set of shift factors, **a**_*T*_, as shown in the WLF plot, adequately shift both storage and loss moduli to consistent, smooth full TTS sweep from the rubbery regime to the glassy regime. The WLF constants obtained for VN01 were approximately *C*_1_ = 42.9 and C_2_ = 257.7.

## Usage Notes

This section provides an overview of data usage. More comprehensive documentation and notes are included in the respective experiment sub-directories and/or by contacting the authors if additional support is needed. To improve the FAIRness, throughout the datasets included in the framework the final output data are converted from raw instrument outputs to accessible, interoperable file formats, and data import and analysis codes are provided to maximize reuse of the data.

### Data usage

#### Micro-computed tomography

The images from μCT are included in raw (projection) format as well as several pre-processed formats. Raw projection images (.txrm), with drift correction data (.txrm) can be used the in the proprietary reconstruction software “Scout-and-scan reconstructor” (see Methods) to re-create the tomographic reconstructions using the recipe settings in the included recipe (.rcp) files. The grayscale 3D reconstruction images are provided as .tif or .tiff image series (one image per *z*-slice, the two extensions refer to the same format). To process the reconstructed 3D images, the supporting analyses directory gives example workflows for binarization (sometimes called segmentation or thresholding) of the reconstruction, and subsequently, visualizing the 3D data and computing relative density. The outputs in the form of binary images (.tif), relative density reports (.csv), and 3D visualization files (.vtk, i.e., unstructured VTK format) are provided in their respective labeled directories and sub-directories. Other macroscopic parameters such as material anisotropy may be measured from the grayscale reconstruction as required. Baseline scans are intended to maximize image quality and serve as the basis for image-based mechanical modeling, e.g., by enabling creation of a 3D finite element mesh from the voxel binary images (.vtk). The high-quality scans may be more suitable for computing metrics for anisotropy, pore connectivity, model transport or flow/diffusion properties of the material, for example via PuMA^35^) or the anisotropy analysis of^17^, or for modeling efforts that consider entrapped gas^36^, since fine structures are more likely to be accurately reconstructed. Compression image stacks (in “COMP” sub-directories) can be used for tasks that have less strict requirements for resolving fine features. For example, we have used digital volume correlation packages such as ALDVC^37^ to measure and correlate overall applied deformation and identify regions of localized (ligament-scale) deformations such as hinging or wall buckling. Depending on the prospective use of the data, the user has direct access to the pre-processed binary images, or may reprocess the grayscale images using different settings in the included analysis scripts, or develop custom segmentation routines. For instance, if accurate cell-wall connectivity is more desirable than accurate relative density, one might lower the threshold (i.e., permit darker pixels to be considered “material”, since thin cell walls tend to be faint compared to noisy void space), and thus reprocess the image stacks accordingly.

#### UTS and digital image correlation

The force, time, and crosshead displacement data from the UTS are stored in .csv tabular data files, which are straightforwardly machine-readable. Images are stored as .tiff (quasi-static) or .tif (intermediate) time-series. For the high-speed camera (intermediate rate configuration) imaging metadata are stamped in the top portion of the image, which can be manually entered into a post-processing routine or interpreted via optical character recognition as needed. Reference length scale images for each dataset are used to convert the pixel lengths to physical units using a virtual ruler tool, for example using the “Measure” analysis in FIJI^38^. Image and load frame data can be processed to compute, for example, stress-strain curves, strain-time curves, and displacement or strain maps, as shown in Figs. 3 and 4 using the qDIC-based^32^ workflow included in the “supporting_analyses” sub-directory. Output data from these analyses are included in respective results (.mat) container files (accessible by many HDF5 readers). Use of other DIC codes on the image series or additional analysis is encouraged and can be applied on the raw data directly or re-use data extractor methods provided with the qDIC implementation. Example usage of stress-strain data from full-field measurements across multiple strain rates includes constitutive model development^9,39^, comparison of direct simulation to experimental data^40^, or impact protection equipment design^13^.

#### Dynamic mechanical analysis

The native instrument file format (.tri) is used for raw data from the DMA experiments. These files contain richer metadata than the respective spreadsheet (.xls) data files that constitute the results from each individual experiment in the series of frequency sweeps used for time-temperature superposition analysis. The raw data are equivalent. Experiment configuration metadata are exported to plain text (.txt). The .tri files can be opened and used in the software “Trios” version 5.1.1 that, at the time of publication, is provided free-of-charge by the instrument manufacturer (see: https://www.tainstruments.com/support/software-downloads-support/downloads/). The preliminary experiments and procedures that were used to calibrate the frequency and temperature range and thermal soak times are in a “preliminary_experiments” folder. Example procedure files that were used to control the instrument are also included in a “Procedures” sub-directory in their native .tprc file format, which can be directly loaded and re-used via the Trios software. Similarly to TTS experiments, both raw instrument format and equivalent interoperable .xls and .txt files are available to use. For building shifted full TTS curves we also include in the supporting analyses sub-directory a Python script (“dma_to_TTS_worksheet.py”) to organize exported data and a pre-programmed spreadsheet to which data are then transferred for manual determination of shift factors and full TTS curve generation. The complete, shifted storage, loss moduli and loss tangents could, for example, be used to inform design guidelines or material selection^13^, or as input to viscoelastic material modeling^41^.

### Data interfaces

#### NIST PDR interface

The NIST PDR version of the dataset^33^ interface provides the user with an entry point to access and download either individual files or multiple files or directories as a *Data Cart*. If multiple files are selected they are automatically compressed into a .zip file container. The landing page shows basic information about the dataset, including a summary abstract, keywords, author information, and other metadata. The metadata are accessible via the JSON-like machine-readable NERDm format common to NIST-published datasets. The data are accessed via a tree table user interface (UI) following the structure described in the Data usage section, a summary of which is given as an entity relationship diagram (foam_data_entity_relationship_diagram.pdf) in the top level of the dataset. The table contains columns for the file or folder name, the type of data, the size, and download status – either via direct download for individual files or via the add-to-cart feature for multiple files or complete directories.

#### MDF Globus interface

A basic interface into the MDF dataset^34^ is provided via the Globus system^42^, which provides a web interface as well as Python and command line interfaces, by which a user can select and directly download individual records (files or data). For robust, bulk file transfers via the web interface users can install a freely available (Apache 2.0 license) Globus Personal endpoint. The Globus web interface provides a two-panel UI similar to many file transfer utilities, with one panel for the data collection on the server and a second for the local endpoint. For more details refer to Globus and related documentation (see: https://docs.globus.org). Examples of data access, filtering, and visualization via Python scripting that access the Globus data are provided and documented as Jupyter notebooks in a Git repository (see: https://github.com/materials-data-facility/foam_db). This scripting interface helps improve access and reuse of data by providing direct examples within common Python data handling routines, such as reading data into “NumPy” arrays and plotting with “matplotlib”. In addition, the repository has documentation, framework structure visualization, brief notes on data usage, and provides a public forum for reporting issues and asking questions about the data and data framework within a formal version-controlled context.

## Data Availability

A snapshot version of our custom analysis tools at the time of initial data preparation is included in the “supporting_analyses” directories within each experiment type. These include μCT (Python), DIC (Matlab), and DMA (Python and Excel) analyses, with documentation. Settings used for μCT analysis are summarized in Table 2 and settings for DIC analysis are in Table 3. All first-party data and code referenced here are available free and open source per the included NIST license file; however, certain commercial formats and analysis software are also used. Example code for access, visualization, and filtering of data are provided free and open source (MIT License) as a Git repository, as described in the *Data interfaces* section.
